# Chondrosarcoma of the Alveolar Process of the Mandible Initially Suspected to Be a Periodontal Lesion

**DOI:** 10.3390/diagnostics14040348

**Published:** 2024-02-06

**Authors:** Biljana Markovic Vasiljkovic, Aleksa Janovic, Svetlana Antic, Branko Dozic, Milos Bracanovic, Djurdja Bracanovic

**Affiliations:** 1 Department of Radiology, School of Dental Medicine, University of Belgrade, 11000 Belgrade, Serbia; biljana.markovic.vasiljkovic@stomf.bg.ac.rs (B.M.V.);; 2 Department of Pathology, School of Dental Medicine, University of Belgrade, 11000 Belgrade, Serbia; branko.dozic@stomf.bg.ac.rs; 3 Clinic for Emergency Surgery, University Clinical Center of Serbia, 11000 Belgrade, Serbia; milosbracanovic@gmail.com

**Keywords:** chondrosarcoma, mandible, periodontal lesion, CBCT, CT

## Abstract

Chondrosarcoma (CS) initially suspected to be a periodontal lesion is atypical and rare. To the best of our knowledge, only six similar cases have been reported so far. A 47-year-old woman presented with a discreet swelling of the alveolar process of the mandible, while adjacent mucosa appeared normal. Upon initial intraoral radiography, a periodontal lesion was suspected by the ordinating dentist. Further radiological evaluations included CBCT, CT, and MRI, which showed a thickening of the supporting bone with ground-glass foci but without visible calcifications. The periodontal space of the affected teeth appeared to be uniformly widened. The destruction of the vestibular and lingual cortex was observed, as well as a discreet periosteal reaction, implying the secondary involvement of these teeth and not the odontogenic nature of the lesion. The lesion was restricted to the alveolar process of the mandible, and the bone marrow was not affected. Upon biopsy, a preliminary histopathology report suggested chondrosarcoma, and the patient underwent surgery. It is important to emphasize the possible malignant nature of atypical lesions in the alveolar bone, especially in cases with the expansion of vestibular and lingual cortical plates. Additionally, postoperative “watch and see” follow-ups may be considered in cases of CS in the jaws.

**Figure 1:**
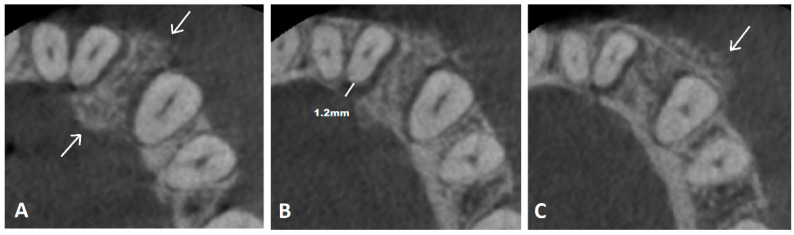
A 47-year-old woman presented with a discreet swelling of the frontal region of the mandible. The swelling was observed 6–8 weeks prior to the initial clinical examination, gradually increasing in size over this period but without any associated symptoms. The patient did not report any prior trauma or other significant bone-related, endocrinological, personal, or family medical history. A clinical exam reported a slight bulging of both the vestibular and lingual aspects of the alveolar process of the mandible in the region of the lower left canine and lateral incisor. The displacement of these teeth caused a visible diastema but without signs of supraeruption. The affected teeth were not mobile. Adherent mucosa appeared normal. Upon initial intraoral radiography, a uniform widening of the periodontal space of the affected teeth was detected, suggesting a periapical lesion. Given this discrepancy between the clinical and radiographic findings, a CBCT was indicated by the ordinating dentist. The CBCT showed thickening of the supporting bone between the lower left canine and lateral incisor and an altered bone structure with zones of ground-glass appearance (white arrows) (**A**). The periodontal space of these teeth appeared to be uniformly widened up to 1.2 mm in width (**B**). Hereupon, our radiological department was consulted. The vestibular and lingual cortex of the alveolar process were destroyed with an obvious bulging of the lesion and a periosteal reaction (white arrow) (**C**). A periosteal reaction was seen on both the vestibular and lingual cortexes, which was more prominent anterior to the canine (**A**,**C**). A malignant process was suspected, implying the secondary involvement of teeth instead of the initially suspected odontogenic nature of the lesion. Additionally, in this case, the affected teeth appeared normal; there were no signs of root canal infection; the periodontal space was uniformly widened (not just in the periapical region); and the lesion showed an expansive–destructive pattern of growth. Malignant bone lesions in the jaws may lead to the loosening and displacement of the affected teeth and the widening of the periodontal space [1,2,3], all of which were evident in our patient as well.

**Figure 2:**
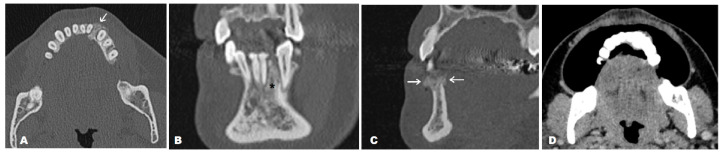
Given the limitations of the CBCT method, a CT scan of the mandible was obtained in both unenhanced and contrast-enhanced (CE) series, primarily in order to evaluate the bone structure of the entire mandible, surrounding soft tissues, and the possible extraoseal propagation of the lesion. A CE scan was performed using the puffed-cheek technique. The lesion was restricted to the alveolar process of the mandible (white arrow) (**A**), apically slightly exceeding the apexes of the left lower canine and lateral incisor (asterisk) (**B**). The size of the lesion was 11.6 mm in vestibule–oral diameter, 10.1 mm in lateral diameter, and 14.9 mm in craniocaudal diameter. There were no signs of root resorption – of the affected teeth. Oblique sagittal reconstruction in the bone window confirmed the presence of a periosteal reaction on both cortical plates (white arrows) (**C**). The CECT showed no enhancement of the adjacent mucosa or surrounding soft tissues (**D**). After a biopsy, preliminary histopathology reported a neoplasm with chondroid differentiation, mild cellular atypia, and a focus of chondroid tissue calcification but without osteoid deposition, suggesting chondrosarcoma (CS). CS initially suspected to be a periodontal lesion is atypical and rare. To the best of our knowledge, only six similar cases have been reported so far [1,3,4,5,6]. The absence of a typical lytic growth pattern and visible calcifications in this case could be explained by the relatively small size of the tumor, approximately 1 centimeter in the greatest diameter. The irregularity of the borders primarily implied protrusive growth but without extension into the nearby structures.

**Figure 3:**
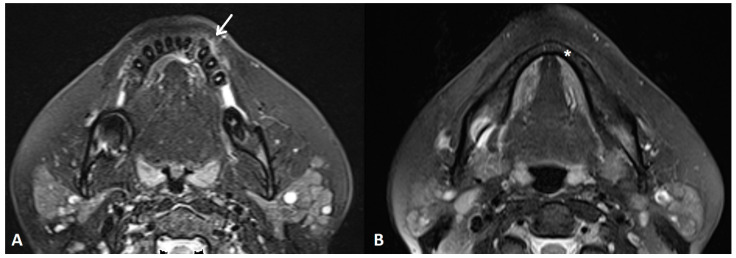
An MRI T2W FS sequence demonstrated a bone lesion in the reported area with irregular focuses of the chondroid/osteoid matrix with non-homogenous signal intensity and a visible periosteal reaction (white arrow) (**A**). There was no alteration of the bone marrow signal intensity in the mandible (asterisk) (**B**). The patient underwent left marginal resection of the mandible, extending from the region of the first molar posteriorly to the medial aspect of the right lower lateral incisor medially. Postoperative histopathology diagnosed the tumor as conventional CS, grade II, with positive resection margins. Therefore, the multidisciplinary team (MDT) suggested oncological follow-up every three months, at least for the first year.

**Figure 4:**
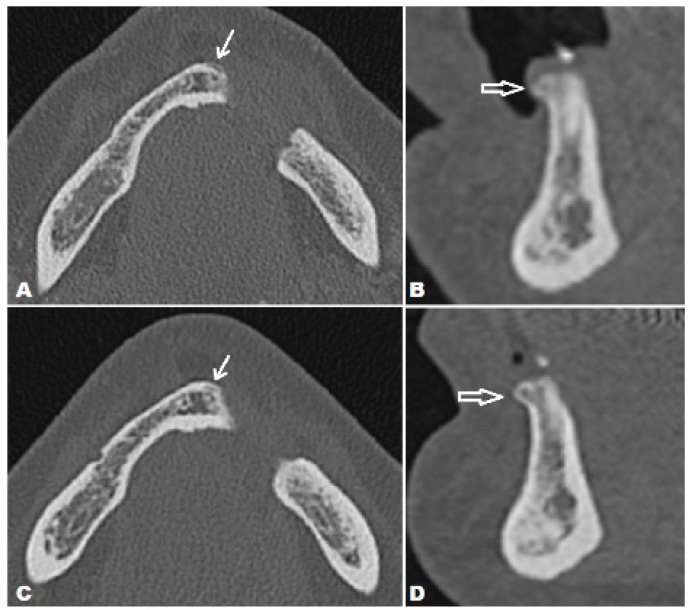
The first postoperative CT 3 months after the surgery showed discreet vestibular periosteal scalloping on the medial resection margin, which is not strictly indicative of residual tumor tissue but needed further evaluation (white arrow) (**A**), (empty white arrow) (**B**). Fortunately, consecutive CT controls showed no alteration in the size or morphology of these lesions, as did MRI controls. Axial (**C**) and oblique sagittal (**D**) CT images showed no progression 12 months after surgery (white arrow) (**C**), (empty white arrow) (**D**). So far, a 2-year follow-up showed no signs of tumor recurrence. Head and neck CS prognosis is based on the tumor location and possibility of radical surgery, size, histological subtype, and grade [1,7,8]. Surgery is by far the most effective therapeutic modality and can be a curative procedure [7,8,9]. Wide excision with a tumor-free margin of 2–3 cm is preferable [10] but not easily achievable, especially in the head and neck region, because of site specificity. Thus, a positive surgical margin, as in the case we have reported, is not surprising. Additionally, as stated in the literature, the recurrence rate of approximately 40% is primarily the result of incomplete surgical resections [8,11]. Having in mind the tumor site and grade, performed surgery, and the questionable effects of radiotherapy and chemotherapy, the decision of the MDT to schedule the patient for a “watch and see” follow-up showed a satisfying result since the patient has been without a detectable recurrence for 2 years.

## Data Availability

The data are contained within the main text of the manuscript.
